# The adaptor proteins HAP1a and GRIP1 collaborate to activate the kinesin-1 isoform KIF5C

**DOI:** 10.1242/jcs.215822

**Published:** 2019-12-13

**Authors:** Alison E. Twelvetrees, Flavie Lesept, Erika L. F. Holzbaur, Josef T. Kittler

**Affiliations:** 1 Sheffield Institute for Translational Neuroscience, Department of Neuroscience, University of Sheffield, Sheffield S10 2HQ, UK; 2 Department of Neuroscience, Physiology and Pharmacology, University College London, Gower Street, London WC1E 6BT, UK; 3 Department of Physiology, Perelman School of Medicine, University of Pennsylvania, Philadelphia, PA 19104-6085, USA

**Keywords:** Kinesin, Molecular motor, Microtubule transport, Autoinhibition, Adaptor proteins

## Abstract

Binding of motor proteins to cellular cargoes is regulated by adaptor proteins. HAP1 and GRIP1 are kinesin-1 adaptors that have been implicated individually in the transport of vesicular cargoes in the dendrites of neurons. We find that HAP1a and GRIP1 form a protein complex in the brain, and co-operate to activate the kinesin-1 subunit KIF5C *in vitro*. Based upon this co-operative activation of kinesin-1, we propose a modification to the kinesin activation model that incorporates stabilisation of the central hinge region known to be critical to autoinhibition of kinesin-1.

## Abstract (precis)

**Summary:** The adaptor proteins HAP1a and GRIP1 form a protein complex in the brain, and co-operate to activate the kinesin-1 subunit KIF5C *in vitro*.

## INTRODUCTION

Motor proteins perform the mechanical work of cellular transport systems, which are key components of how cells function and modify their behaviour. Many advances have been made in understanding the chemomechanical mechanisms of force generation by motor proteins (Carter et al., 2016; Hancock, 2016), but critical questions remain about how motors work within cells.

Kinesins are microtubule motors that move towards the plus ends of microtubules. The archetypal kinesin, kinesin-1, is formed of two heavy chains (KIF5A–KIF5C) and two light chains (KLC1–KLC4), with no apparent preference of heavy chains for particular light chains. Significant evidence states that kinesin-1, when not bound to cargo, exists in a folded autoinhibited conformation (Coy et al., 1999; Friedman and Vale, 1999; Stock et al., 1999). Autoinhibition is mediated by direct interactions between the head and tail of the KIF5 (Coy et al., 1999; Kaan et al., 2011) and a central hinge that allows folding (Friedman and Vale, 1999). KLCs also contribute to both the inhibition of the motor activity of the kinesin (Blasius et al., 2007; Verhey et al., 1998) and the activation of the motor by cargo through release of KLC autoinhibition (Yip et al., 2016). In order to activate kinesin, current models suggest that the head–tail KIF5 interaction must be overcome (Kaan et al., 2011), the KLC block must be removed (Blasius et al., 2007; Verhey et al., 1998; Yip et al., 2016), and adaptor proteins must bind to the ‘cargo-binding domain’ (CBD) of KIF5 (Blasius et al., 2007; Cho et al., 2009). Many proteins that bind to the KIF5 CBD have been identified for specific cellular cargoes (see Seeger and Rice, 2013 for a summary). Adaptor protein specificity underlies the ability of a small number of motor proteins to transport many unique cargoes. However, direct activation of KIF5 by adaptor proteins has only been characterised in a very few cases (Blasius et al., 2007; Cho et al., 2009; Sun et al., 2011), leaving many questions about how these findings relate to other structurally diverse, multi-component transport complexes.

Two kinesin adaptor proteins critical for neuronal function are glutamate receptor interacting protein 1 (GRIP1) and huntingtin-associated protein 1 (HAP1). GRIP1 was identified as an adaptor linking excitatory α-amino-3-hydroxy-5-methyl-4-isoxazolepropionic acid receptors (AMPARs) to KIF5 for their delivery to excitatory synapses (Setou et al., 2002). It has subsequently been shown to be important for the trafficking of the transmembrane proteins EphB receptors (Hoogenraad et al., 2005) and N-cadherin (Heisler et al., 2014) in dendrites. We have shown previously that HAP1 is an adaptor between inhibitory γ-amino-butyric acid type A receptors (GABA_A_Rs) and KIF5 necessary for the recycling of receptors back to the surface of dendrites (Twelvetrees et al., 2010). In addition, HAP1 is involved in trafficking other neuronal transmembrane proteins, including: the amyloid precursor protein (McGuire et al., 2006; Yang et al., 2012), the neurotrophin receptors TrkA, TrkB and p75^NTR^ (also known as NTRK1, NTRK2 and NGFR, respectively) (Lim et al., 2017; Rong et al., 2006), and epidermal growth factor receptors (Li et al., 2003). HAP1 is also essential for the trafficking of BDNF at several stages of its life cycle (Gauthier et al., 2004; Lim et al., 2017; Wu et al., 2010; Yang et al., 2011).

Critically, however, there is currently no direct evidence that either GRIP1 or HAP1 can independently activate kinesin-1 motors to facilitate transport. Furthermore, despite overlapping roles in linking cargo to kinesins for dendritic neuronal transport, the interplay between GRIP1 and HAP1 has not been studied. Here, we report that GRIP1 and HAP1 form an endogenous kinesin-activating complex by binding distinct sites on the KIF5C polypeptide. Using *in vitro* studies, we demonstrate that HAP1 and GRIP1 work together to activate kinesin. Subsequently, we propose that kinesin activation may include stabilisation of the hinge region to prevent folding of KIF5.

## RESULTS AND DISCUSSION

### GRIP1 and HAP1a form a complex endogenously

**Fig. 1.**
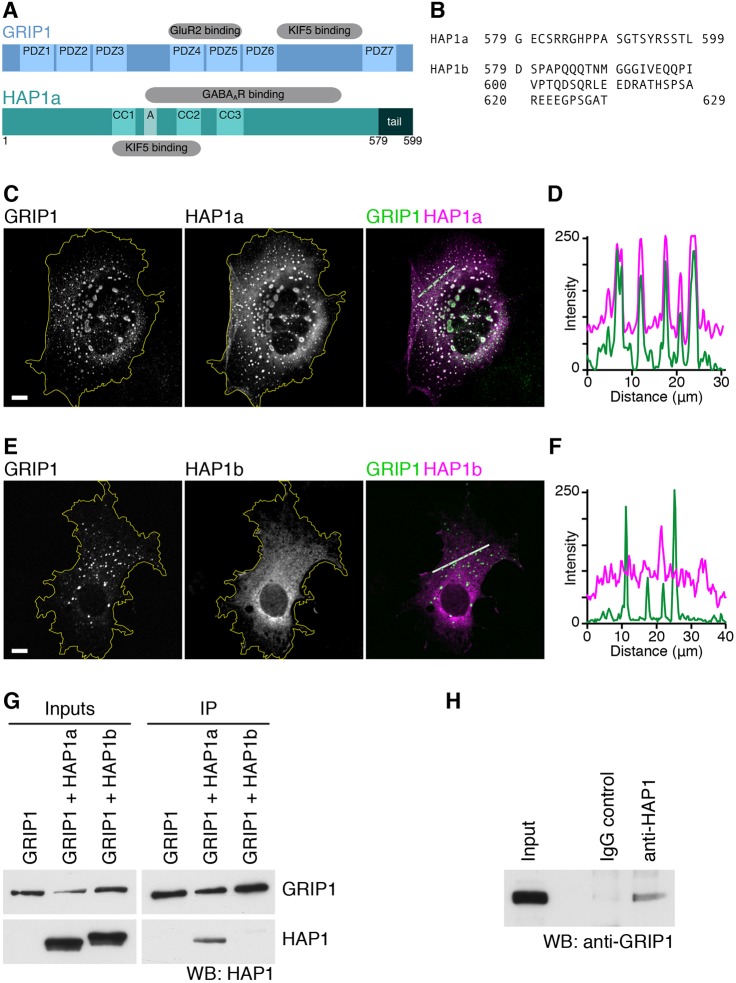
**GRIP1 and HAP1 form a complex in cells and in brain.** (A) Schematic of GRIP1 and HAP1 domains. PDZ, PDZ domain; CC, coiled-coil; A, acidic domain; tail, variable C-terminal tail. (B) C-terminal sequences of rat HAP1a and HAP1b. (C–F) COS cells co-transfected with GFP–GRIP1a and HA–HAP1a show recruitment of GRIP1 to HAP1a puncta (C,E). Yellow line, cell periphery. Scale bars: 10 μm. (D,F) Line scans through the merged images at the section highlighted with the white line; peaks correspond to punctate structures. (G) Western blot (WB) of immunoprecipitation from COS cells co-transfected with GFP–GRIP1a and either HAP1a or HAP1b, immunoprecipitated with anti-GFP antibody. The interaction is specific to HAP1a. (H) Western blot of GRIP1 co-immunoprecipitated with HAP1 from rat brain homogenate.

**Fig. 2.**
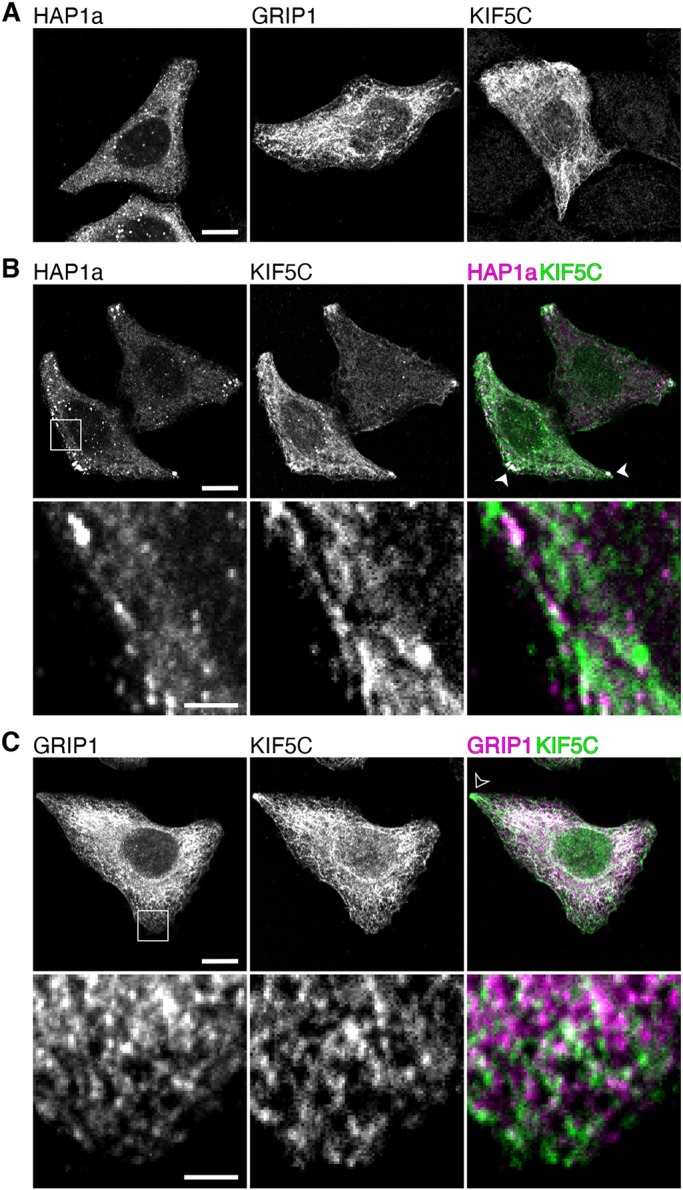
**HAP1a but not GRIP1 redistributes to the periphery of HeLa cells with KIF5C.** (A) Singly transfected HeLa cells showing the distribution of HAP1a, GRIP1 and KIF5C, respectively. Scale bar: 10 µm. (B) KIF5C recruits HAP1a to the periphery of co-transfected HeLa cells, highlighted by white arrowhead. An enlarged area shows superposition of HAP1a and KIF5C puncta. Scale bars: 10 μm (main image) and 2 μm (enlargement). (C) KIF5C is unable to recruit GRIP1 to the periphery of co-transfected HeLa cells. The black arrowhead highlights KIF5C-positive GRIP1-negative peripheral puncta. Scale bars: 10 μm (main image) and 2 μm (enlargement).

There are two isoforms of HAP1 in rodents, HAP1a and HAP1b, which are identical over the first 578 residues with differing C-terminal ‘tail’ sequences (Fig. 1A,B). HAP1a, but not HAP1b, has a potential C-terminal type I PDZ domain ligand motif that could bind the PDZ domains of GRIP1 (Ye et al., 2000). GRIP1 undergoes alternative splicing at the N-terminus to generate GRIP1a and GRIP1b. To investigate the possibility of a protein–protein interaction between GRIP1 and HAP1, we performed immunofluorescence screening in co-transfected COS cells (Fig. 1C–F). HAP1a and GRIP1a both form puncta when expressed in cell lines (see Fig. 2A for singly transfected cells). Puncta are likely related to an endogenous non-membrane-bound organelle formed by HAP1; within the hypothalamus, HAP1 is highly expressed (Chan et al., 2002; Li et al., 2003; Sheng et al., 2006) and associated with non-membrane-bound cytoplasmic bodies (Li et al., 1998; Shinoda et al., 1992, 1993; Xiang et al., 2017) that sequester several key proteins in culture (Prigge and Schmidt, 2007; Rong et al., 2007; Sheng et al., 2008; Takeshita et al., 2011, 2006). When co-expressed in the same cells, GRIP1a and HAP1a are recruited to the same intracellular compartment (Fig. 1C,D). In contrast, HAP1b has a diffuse cytosolic distribution and does not overlap with GRIP1a (Fig. 1E,F). As opposed to full-length GRIP1a, PDZ domains 4–6 of GRIP1 (GRIP1-PDZ456) have a diffuse cytosolic distribution when expressed in COS cells, but are recruited to puncta when co-expressed with HAP1a (Fig. S1).

In co-immunoprecipitations (co-IPs) from COS cells co-transfected with GFP–GRIP1a and HA-tagged HAP1a or HAP1b (HA–HAP1a or HA–HAP1b), anti-GFP could co-IP HAP1a, but not HAP1b (Fig. 1G). This confirmed that the interaction is mediated by the 19 amino acids of the HAP1a tail. Finally, in order to establish whether GRIP1 and HAP1 form an endogenous complex, we performed co-IPs from rat brain homogenate. A co-IP performed using antibodies for HAP1 that we have previously shown readily co-immunoprecipitate KIF5 (Twelvetrees et al., 2010) also co-immunoprecipitated GRIP1 (Fig. 1H).

### HAP1a but not GRIP1 is trafficked by KIF5C to the cell periphery

Having observed co-recruitment by immunofluorescence in COS cells overexpressing GRIP1 and HAP1a, we used immunofluorescence to compare HAP1 and GRIP1 interactions with KIF5 isoforms.

We demonstrated previously that HAP1a and KIF5 proteins interact through the KIF5 CBD (Twelvetrees et al., 2010). When KIF5C is overexpressed in COS cells, it has a tendency to accumulate in the cell periphery (Dunn et al., 2008). Consistent with these observations, when overexpressing full-length KIF5C with HAP1a in HeLa cells or COS cells, we saw good overlap and a pronounced shift in the localisation of HAP1a clusters away from the perinuclear region and into the periphery (Fig. 2; Fig. S2). Similar results were observed with KIF5B, but not KIF5A (Fig. S2), mirroring our previous data showing that HAP1a interacts poorly with KIF5A compared to KIF5B and KIF5C *in vitro* (Twelvetrees et al., 2010).

In contrast, we saw little overlap between GRIP1 and KIF5C by immunofluorescence in co-transfected HeLa cells or COS cells and no GRIP1 in the cell periphery (Fig. 2; Fig. S2). This observation was true for all three KIF5 isoforms (Fig. S2). In overexpression studies in COS cells, we were also unable to observe an interaction between full-length KIF5 and GRIP1 by co-immunoprecipitation (data not shown).

### Co-expression of HAP1a allows the KIF5C-mediated redistribution of GRIP1 to the cell periphery

**Fig. 3.**
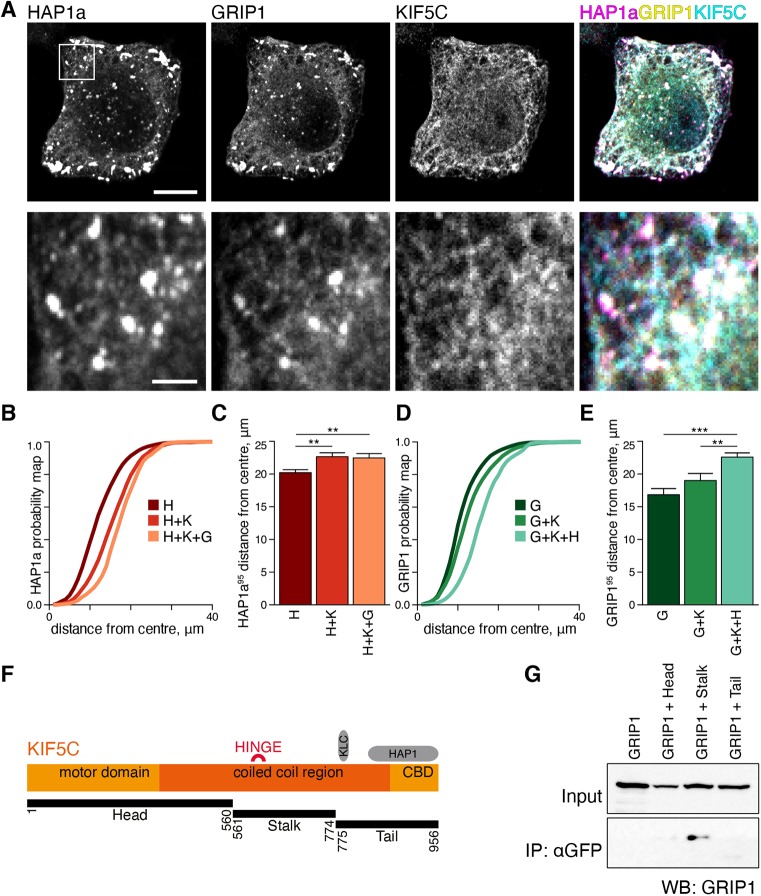
**GRIP1 can co-complex with KIF5C in the presence of HAP1a through the KIF5 stalk.** (A) KIF5C is able to recruit GRIP1 to the periphery of co-transfected HeLa cells when HAP1a is also present. The boxed area is enlarged on bottom row. Scale bars: 10 μm (main image) and 2 μm (enlargement). (B) Plot of cumulative distribution of HAP1a signal according to distance from the centre of a cell (HAP1a probability map). Displacement to the right compared to that in HAP1a only (denoted H) indicates that the HAP1a signal is accumulated further from the centre of the cell. H+K, HAP1a plus KIF5C; H+K+G, HAP1a plus KIF5C and GRIP1a. Analysis was performed from three independent experiments (*n*=number of cells; in H, 53; H+K, 43; H+K+G, 39). (C) The distance from the cell centre at which 95% of the HAP1a signal is found. Analysis was performed from three independent experiments (*n*=number of cells; in H, 53; H+K, 43; H+K+G, 39). **P<0.01 (one-way ANOVA test). (D) Plot of the cumulative distribution of GRIP1a signal according to distance from the centre of a cell (GRIP1a probability map). Displacement to the right compared to GRIP1a only (denoted G) indicates that GRIP1a signal is accumulated further from the centre of the cell. G+K, GRIP1a plus KIF5C; G+K+H, GRIP1a plus KIF5C and HAP1a. Analysis was performed from three independent experiments (*n*=number of cells; in G, 31; GK, 29; GKH, 35). (E) The distance from the cell centre at which 95% of the GRIP1a signal is found. Analysis was performed from three independent experiments (*n*=number of cells in G, 31; G+K, 29; G+K+H, 35). **P<0.01; ***P<0.001 (one-way ANOVA test). (F) Schematic representation of KIF5 polypeptide chain showing functional regions and constructs used. (G) Western blot of co-IP from COS cells showing that Myc–GRIP1 preferentially binds to the stalk region of KIF5.

Given the interaction of HAP1a with GRIP1 and KIF5C when co-expressed in cells, we speculated that the addition of HAP1a would increase the overlap of GRIP1 with KIF5C. Cells co-transfected with GRIP1, HAP1a and KIF5C show good overlap of GRIP1 with KIF5C and redistribution of GRIP1 to the periphery of the cell (Fig. 3A; Fig. S3E,F). To quantify the redistribution of GRIP1 and HAP1a to the cell periphery by KIF5C, we performed Sholl analysis on transiently transfected HeLa cells. HAP1a showed a significant shift to the cell periphery in the presence of KIF5C, which was not enhanced by the addition of GRIP1 (mean±s.e.m. distance from the centre 20.21±0.46, 22.66±0.61 and 22.46±0.67 for HAP1a only, KIF5C+HAP1a or GRIP1+KIF5C+HAP1a, respectively; Fig. 3A–C). In contrast, GRIP1 localisation was not changed in the presence of KIF5C alone, but did show a pronounced shift with the addition of HAP1a (mean±s.e.m. distance from the centre 16.86±0.93, 19.02±1.07, 22.60±0.65 for GRIP1 only, KIF5C+GRIP1 or HAP1a+KIF5C+GRIP1, respectively; see Fig. 3D,E). Both KIF5B and KIF5C interact well with HAP1a, whereas the interaction with KIF5A is relatively weak (Twelvetrees et al., 2010). Consequently, in cells triple transfected with KIF5A, GRIP1 and HAP1a, only the signal for GRIP1, and HAP1a overlap with one another and KIF5A itself is not found within the HAP1a and GRIP1 double-positive puncta (Fig. S3A,B), contrary to observations with KIF5B and KIF5C (Fig. S3C–F).

Biochemical and biophysical studies support the model that the majority of overexpressed KIF5 in our COS cell system should be in a folded auto-inhibited conformation due to the lack of similarly overexpressed adaptor proteins (Coy et al., 1999; Friedman and Vale, 1999; Stock et al., 1999). We speculated that the GRIP1-binding site on KIF5 might be masked when KIF5 is autoinhibited and only exposed in the presence of HAP1a if HAP1a causes release of KIF5 autoinhibition in a manner similar to JIP1 (also known as MAPK8IP1) (Blasius et al., 2007), JIP3 (also known as MAPK8IP3) (Sun et al., 2011) and RanBP2 (Cho et al., 2009), which bind to the KIF5 CBD. In co-IP studies with KIF5C fragments from transfected COS cells, we were able to recapitulate the interaction between GRIP1 and KIF5C (Fig. 3F,G). Surprisingly, the most efficient GRIP1 interaction occurred with the KIF5C ‘stalk’ region, rather than the KIF5C ‘tail’ that incorporates the KIF5 CBD where most adaptors typically bind.

### GRIP1 and HAP1a are sufficient to activate KIF5C in *in vitro* motility assays

The trafficking of HAP1a to the cell periphery in the presence of KIF5C is suggestive of HAP1a release of KIF5C autoinhibition. Additionally, GRIP1 is unable to associate with kinesin and traffic to the cell periphery without the presence of HAP1a. To test whether GRIP1 needed HAP1a to activate kinesin, we carried out *in vitro* studies to characterise the activation of kinesin in the presence of these adaptor proteins.

**Fig. 4.**
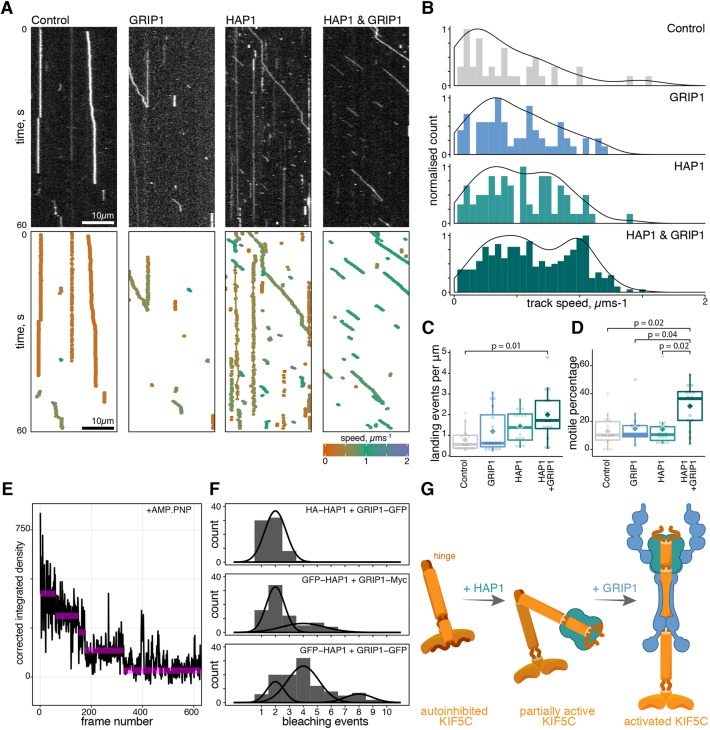
**Activation of KIF5 motility *in vitro* requires both HAP1a and GRIP1.** (A) Representative motility of KIF5C–Halo in the presence of adaptor proteins as shown by kymographs and corresponding tracking data. (B) Histograms of the track speed of motile particles for each condition; *n*=39, 63, 66 and 302 for Control, GRIP1, HAP1a and HAP1a and GRIP1, respectively, from four independent experiments. Histograms are also overlaid with a scaled Gaussian kernel density plot (solid line). (C) Landing events per micrometre of microtubule; *n*=14–16 microtubules from four independent experiments. (D) Motile percentage from the total number of landing events; *n*=14–16 microtubules from four independent experiments. Box plots are presented as described in Materials and Methods. (E) Example trace of AMP-PNP-immobilised GFP puncta fluorescence intensity bleaching over time. (F) Photobleaching events per molecule. Parameters of fitted Gaussians are listed in Tables S1 and S2. (G) Model of KIF5 activation by GRIP1 and HAP1. HAP1a binding is insufficient to stabilise the open confirmation on its own, but upon co-addition of GRIP1, KIF5 is robustly activated.

We analysed the activation of KIF5C by total internal reflection fluorescence microscopy (TIRFM), in a similar manner to that previously described (Blasius et al., 2007; Sun et al., 2011). COS cells were mock transfected for the control condition or with either HA–HAP1a or Myc–GRIP1a. Cell lysates containing individually expressed adaptor proteins were mixed with lysate from cells expressing KIF5C labelled with HaloTag TMR ligand (KIF5C–Halo). Mixing lysates ensured equimolar amounts of KIF5C–Halo in each condition. Mixed lysates were incubated at room temperature, diluted in assay buffer and passed into a flow chamber containing immobilised HiLyte 488-labelled microtubules and imaged by TIRFM (Fig. 4A).

We observed a small number of KIF5C–Halo landing (microtubule binding and release) and motile events in the absence of GRIP1 or HAP1a (Fig. 4A, Control), likely due to endogenous protein interactions from the cell lysate or stochastic activation (Fig. 4B). Adding GRIP1 or HAP1a individually produced more landing events (total landing events: 304, 490 and 652 for Control, GRIP1 only and HAP1a only, respectively; median landings per micrometre of 0.56, 0.62 and 1.38; mean±s.e.m. landings per micrometre of 0.78±0.15, 1.19±0.27, 1.44±0.21; see Fig. 4C) and slightly faster motility (Fig. S4A). Increased landing events produced more KIF5C–Halo motile events (*n*=39, 63, 66 for Control, GRIP1 only and HAP1a only, respectively), although the proportion of landing events that result in motility remained constant (Fig. 4D), indicating stochastic activation of KIF5C once bound to microtubules was the same in all three conditions. As the concentration of KIF5C is the same in all conditions, this may represent partial or transient unfolding caused by exposure of the MT binding site in either the head or the tail of KIF5C (Hackney and Stock, 2000, 2008).

However, a striking difference in KIF5C motility was observed when both GRIP1 and HAP1a were added simultaneously. Not only were there over three times more landing events compared to the Control (total=942; median landings per micrometre of 1.72; mean±s.e.m. landings per micrometre of 2.00±0.3; Fig. 4C), the motility observed following landing displayed increased speed (Fig. 4B; Fig. S4A). Characteristic run lengths were: Control, 2.3±0.07; GRIP1, 3.4±0.08; HAP1a, 2.6±0.2; HAP1a and GRIP1, 2.4±0.03 where ± indicates the standard error of the fit (see also Fig. S4B,C). Critically, it is only in the condition where both HAP1a and GRIP1 are present that there is a large shift in the proportion of landing events that result in successful motility (Fig. 4D, median motile percentage: Control, 10.3%; GRIP1 only, 10.9%; HAP1a only, 10.5%; HAP1a and GRIP1, 36.4%). Analysis of photobleaching steps for motor–adaptor complexes immobilised on microtubules after treatment with the non-hydrolysable ATP analogue AMP-PNP indicated there were likely two GRIP1 molecules and two HAP1a molecules bound per motor (most particles with GFP-labelled HAP1a and GRIP1 had four bleaching steps), although some molecules from the cell lysate likely represented a dimer of dimers (Fig. 4E,F).

Adaptor protein specificity is thought to underlie the ability of a relatively small number of motor proteins to transport many unique cargoes. Consistent with this, substantial evidence suggests both AMPARs (Hoerndli et al., 2013; Hoerndli et al., 2015; Setou et al., 2002) and GABA_A_Rs (Nakajima et al., 2012; Twelvetrees et al., 2010) are delivered to distinct postsynaptic sites (Gu et al., 2016) by KIF5 motors. Prior to this work, the role of adaptors GRIP1 and HAP1 would have fitted neatly into this model. However as crosstalk between the two now seems necessary to activate KIF5, this raises a different possibility. GRIP1 is colocalised at inhibitory synapses both *in vitro* and *in vivo* (Burette et al., 1999; Charych et al., 2006, 2004; Dong et al., 1999a; Kittler et al., 2004a; Li et al., 2005; Wyszynski et al., 1999), and signalling pathways acting on the GRIP1 and HAP1 co-complex (both are phospho-proteins; Kulangara et al., 2007; Rong et al., 2006) could allow crosstalk between excitatory and inhibitory synapses.

Taken together, our data support a role for adaptor binding to additional binding elements along the stalk of KIF5 to promote true motor activation (Fig. 4G). Previous work on one of the first identified kinesin-1 activators, JIP1, has also isolated an interaction with the stalk domain (Fu and Holzbaur, 2013). The many contact points between JIP1 and kinesin may have masked the importance of the stalk interactions within cells (Blasius et al., 2007; Fu and Holzbaur, 2013). KLCs were recently shown to have their own autoinhibition mechanism (Yip et al., 2016) and it is still unclear how KLC and KIF5 function together in cargo recognition and motor activation. As HAP1 also binds KLCs (McGuire et al., 2006) through the conserved KLC-binding motifs (Dodding et al., 2011), HAP1a and GRIP1 are a complementary system to dissect the principles of kinesin-1 tetramer activation.

In conclusion, we show that structurally distinct adaptor proteins can work together to promote full activation of KIF5C in cells. The co-operative activation mechanism employed by GRIP1 and HAP1a relies on HAP1a binding to the KIF5 CBD, and a previously uncharacterised interaction between GRIP1 and the stalk of KIF5, which further promotes kinesin activation possibly through stabilising the central hinge.

## MATERIALS AND METHODS

### Antibodies and constructs

Mouse anti-HAP1 (clone 1/HAP1, cat. no. 611302; 1:250) and mouse anti-GRIP1 (clone 32/GRIP, cat. no. 611319; 1:200) were both from BD Biosciences. Fluorescent secondary antibodies were from Invitrogen; HRP-conjugated secondary antibodies were from Rockland.

All constructs used have been previously described: GFP–GRIP1a (Hanley and Henley, 2010); Myc–GRIP1a (Kittler et al., 2004a); Myc–GRIP1–PDZ456 (residues 435–969) (Dong et al., 1999b); HA–HAP1a and HA–HAP1b (Kittler et al., 2004b; Li et al., 1995); Myc–KIF5A, Myc–KIF5B and Myc–KIF5C (Twelvetrees et al., 2010); KIF5C-Head–GFP, Stalk–GFP and Tail–GFP, and KIF5C-Halo (Twelvetrees et al., 2016).

### co-IPs using rat brain homogenate

Co-IPs in rat brain homogenate was performed as previously described (Twelvetrees et al., 2010). All animal experiments were performed according to approved guidelines (schedule 1 procedures).

### COS cell co-IP

COS cells (COS-7 cells from ATCC) were cultured in Dulbecco's modified Eagle's medium (DMEM, GIBCO), supplemented with 10% heat inactivated fetal bovine serum (FBS) and penicillin-streptomycin in a humidified 5% CO_2_ atmosphere at 37°C. Cells were transfected using the Amaxa Nucleofector device (Lonza) following the manufacturer's protocol. Transfected cells were harvested at 24 h post transfection. 10 cm dishes of COS cells were solubilised in 0.5 ml of IP buffer (50 mM Tris-HCl pH 7.5, 0.5% Triton X-100, 150 mM NaCl, 1 mM EDTA, 1 mM PMSF in the presence of antipain, pepstatin and leupeptin) for 10 min at 4°C. Detergent-solubilised extracts were collected following centrifugation for 10 min at 17,900 ***g*** at 4°C, placed in a fresh 1.5 ml microcentrifuge tube and incubated with 1 µg of antibody for 1 h. Complexes were precipitated with 15 µl of Protein G–Sepharose beads. Beads were washed three times with IP buffer then resuspended in 3× protein sample buffer and analysed by SDS-PAGE and western blotting. 0.5% input was loaded as a comparison.

### COS cell immunofluorescence

COS cells were fixed by incubation in −20°C methanol for 10 min. Coverslips were washed three times with PBS then blocked by incubation in block solution (PBS with 10% horse serum, 0.5% BSA also containing 0.2% Triton X-100) for 10 min. Primary and secondary antibodies were diluted in block solution and incubated with coverslips for 1 h at room temperature, with six brief washes of PBS between incubations. Coverslips were mounted onto low iron, clear glass slides using ProLong Gold antifade reagent (Invitrogen) and sealed with nail varnish. Samples were imaged by confocal laser-scanning microscopy (CLSM) using a Zeiss LSM 510 META confocal microscope. All images were digitally captured with LSM software with excitation at 488 nm for GFP and Alexa Fluor 488, 568 nm for Alexa Fluor 543 and 633 nm for Cy5-conjugated secondary antibodies. Pinholes were set to 1 Airy unit creating an optical slice of 0.8 μm. Linescans were prepared in ImageJ/FIJI. Images for publication were prepared with ImageJ/FIJI and Adobe Photoshop.

### GRIP1a and HAP1a distribution analysis in HeLa cells

HeLa cells (ATCC) were transfected using the Amaxa Nucleofector device (Lonza) following the manufacturer's protocol. Cell lines were allowed to express the exogenous protein for 24 h before immunochemistry using the same protocol as for COS cells. All cell types were maintained at 37°C in a humidified atmosphere with 5% CO_2_. All confocal images were acquired on a Zeiss LSM700 upright confocal microscope (Carl Zeiss, Welwyn Garden City, UK) using a 63× oil immersion objective (NA 1.4) with 1024×1024 pixels (101 μm×101 μm) resolution. A suitable threshold was selected for each channel and Sholl analysis of GRIP1a and HAP1a HA distribution was performed using a custom-made ImageJ plugin (López-Doménech et al., 2018, 2016). For every analysis, the cell was isolated, removing signal coming from other cells around it. Then the centre of the cell was manually placed and the amount of GRIP1a and HAP1a pixels within shells radiating out from the soma at 1 μm intervals were automatically quantified. The cumulative distribution of GRIP1a and HAP1a signal was plotted as a function of distance from the centre of the cell. The distance where 95% of the total GFP–GRIP1a and HA–HAP1a signal was calculated for each cell by interpolation. One average of the GRIP1^95^ and HAP1a^95^ value was calculated from all experiments performed (*n*=total number of cells from three independent experiments). The experimenter was blind to the experimental conditions for this distribution analysis.

### *In vitro* motility assays

COS cells were chosen for their low amounts of endogenous kinesin-1 (Cai et al., 2007). FuGENE 6 (Promega) was used to transfect COS cells with expression plasmids, following the manufacturer's instructions. All transfections for this assay were single transfections and cell lysates were mixed immediately prior to the experiment (see below). Transfected cells were labelled with HaloTag TMR Ligand (Promega G8252) at 1:10,000 final dilution for 15 min at 37°C, 5% CO_2_. Cells were washed three times with fresh medium at 37°C, 5% CO_2_ for a total of 30 min to remove unbound ligand. Trypsinised cell pellets were washed three times in PBS and lysed in 100 µl of lysis buffer (40 mM HEPES/KOH pH 7.4, 1 mM EDTA, 120 mM NaCl, 0.1% Triton X-100 with protease inhibitor cocktail and 1 mM ATP) for ten minutes on ice. Lysate was cleared by spinning at 18,000 ***g*** at 4°C for 10 min in a benchtop microcentrifuge. Cleared lysate was kept on ice prior to imaging.

1 µl of KIF5C–Halo lysate was mixed with either 10 µl of control lysate, 10 µl of HAP1a lysate, 10 µl of GRIP1a lysate or 10 µl of a 50:50 mix of HAP1a and GRIP1a lysates. Mixtures were incubated for 15 min at room temperature prior to application to the flow chamber (see below). This 10:1 ratio was to ensure saturation of the available kinesin motors with adaptor protein. Activation of kinesin was highly variable in the absence of pre-incubation. Immediately prior to loading into the flow chamber, the lysate mix was diluted 1:20 in P12 buffer (12 mM PIPES, 1 mM EGTA, 2 mM MgCl_2_), of which 1 µl went on to be imaged (see below). Thus, the final dilution of labelled kinesin was 2000 fold, and cell lysate as a whole was 200 fold.

Flow chambers (∼10 µl volume) were assembled from cleaned and silanised (PlusOne Repel-Silane ES, GE Healthcare) coverslips bound on two sides by double-sided tape and vacuum grease to make a flow channel. Flow chambers were prepared by flowing in solutions in the following order: 10 µl of anti β-tubulin (Sigma, TUB2.1) diluted 1:100 in BRB80 (80 mM PIPES, 1 mM MgCl_2_, 1 mM EGTA pH 6.8) with 5 min incubation at room temperature (RT); 10 µl of the blocking reagent 50 mg/ml Pluronic F-127 (Sigma) with 5 min at RT; 20 µl of HiLyte 488 labelled microtubules (1:40 ratio of labelled:unlabelled tubulin; labelled tubulin from Cytoskeleton, Inc.; unlabelled tubulin purified in-house from bovine brain) diluted in TBRB80 (BRB80+20 µM Taxol) with 5 min at RT; 20 µl of P12T (P12+20 µM Taxol) to wash out unbound microtubules. Into this prepared chamber was flowed 1 µl of prepared cell lysate diluted in 10 µl of assay buffer (P12T+0.3 mg/ml BSA, 0.3 mg/ml casein, 10 mM DTT, 10 mM MgATP, 15 mg/ml glucose, 0.5 μg/ml glucose oxidase and 470 U/ml catalase).

Imaging was performed with Ultraview Vox (PerkinElmer) system with 100× apochromat 1.49 NA oil immersion objective (Nikon). Chambers were imaged at 5 frames per second.

### *In vitro* motility analysis

Kinesin particle tracks were analysed using the TrackMate plugin for FIJI/ImageJ (Schindelin et al., 2012). Particle size was ∼0.8 µm or 5 pixels (pixel size=158 nm). Tracks were fitted with sub-pixel resolution. Particles within the TIRF field, but not in close enough proximity to bind to the microtubule, were automatically excluded from analysis.

Track data generated by TrackMate was subsequently analysed in R software with the dplyr package to summarise data and ggplot2 for plotting. Given the unidirectional nature of kinesin tracks on microtubules, track speed was measured as the whole track displacement relative to the track duration. To be included as a ‘landing event’, the particle had to be bound for 0.4 s (two frames), related to the frame rate of 5 fps. To get accurate analysis of track speed, particles that moved less than 0.6 µm were excluded.

Summary data is represented as box and whisker plots where the heavy horizontal line represents the median, box upper and lower limits represent 25th and 75th percentiles and whiskers extend to the closest value within 1.5× the inter-quartile range. The mean is represented by a diamond. Significant difference within data sets was initially tested using the Kruskal–Wallis rank sum test and, if *P*<0.05, was further investigated using pairwise Mann–Whitney–Wilcoxon tests with Bonferroni correction. These tests make no assumptions about the data distribution; values of *P*<0.05 are reported.

Characteristic run length was determined using methods as previously described (Thorn et al., 2000) using the nonlinear least-squares function (nls) in R software. Cumulative frequency distributions (Fig. S4) were compared using the Kolmogorov–Smirnov test.

Photobleaching event analysis was carried out on puncta immobilised on microtubules with 15 mM AMP-PNP in the assay buffer instead of ATP. Briefly, using ImageJ a 6×6 region of interest (ROI) was centred on spots bound to microtubules based on the maximum projection of the image stack, together with an adjacent 6×6 ROI to measure local background and account for uneven illumination. Background was subtracted from the integrated density of each spot at every time point and plotted. Bleaching events corresponding to clear steps in fluorescence intensity were counted for each spot. Population analysis on photobleaching events was performed using model-based clustering with the mixtools package in R software. Given that the number of photobleaching events for any complex must be an integer, fitting was performed specifying that the number of distributions in the data was three, with the mean (μ) of each distribution constrained to 2, 4 and 8 photobleaching events, respectively. The standard deviation (σ) and amplitude (λ) for the resulting distributions is shown in Table S1 and normal distributions plotted in Fig. 4F.

## Supplementary Material

Supplementary information
