# Supplementary material for: The adaptor proteins HAP1a and GRIP1 collaborate to activate the kinesin-1 isoform KIF5C
Source: J Cell Sci. 2019 Dec 13;132(24):jcs215822. doi: 10.1242/jcs.215822 (PMC6955223; doi:10.1242/jcs.215822)
Supplement: Supplementary information [file joces-132-215822-s1.pdf]

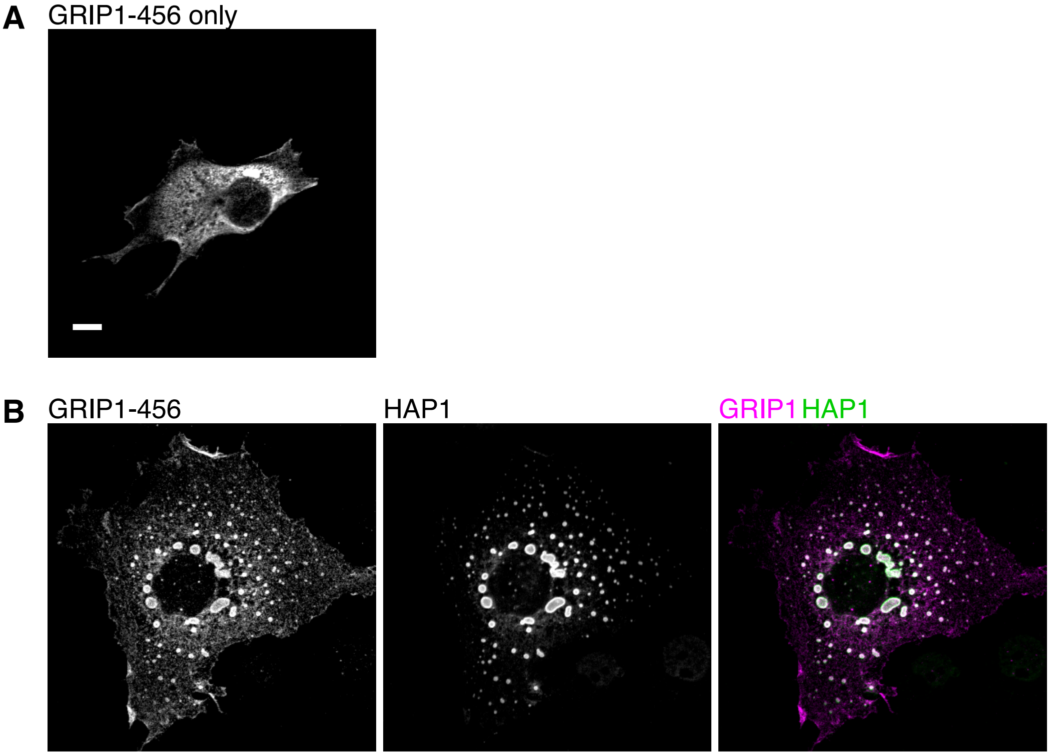

Figure S1

GRIP1-PDZ456 is recruited to HAP1a puncta in COS cells

A COS cells cotransfected with PDZ domain 4-6 of GRIP1 (Myc-GRIP1-PDZ456) only, fixed in methanol and stained with rabbit anti-Myc primary antibodies. GRIP1-PDZ456 shows a diffuse distribution when overexpressed alone. Scale bar = 10µm.

B COS cells cotransfected with Myc-GRIP1-PDZ456 and HAP1a show strong recruitment of GRIP1-PDZ456 to HAP1a puncta. COS cells were transfected with GRIP1-PDZ456 (magenta) alone in combination with HA-HAP1a (green) and fixed in methanol before staining with mouse anti-HA and rabbit anti-Myc primary antibodies.

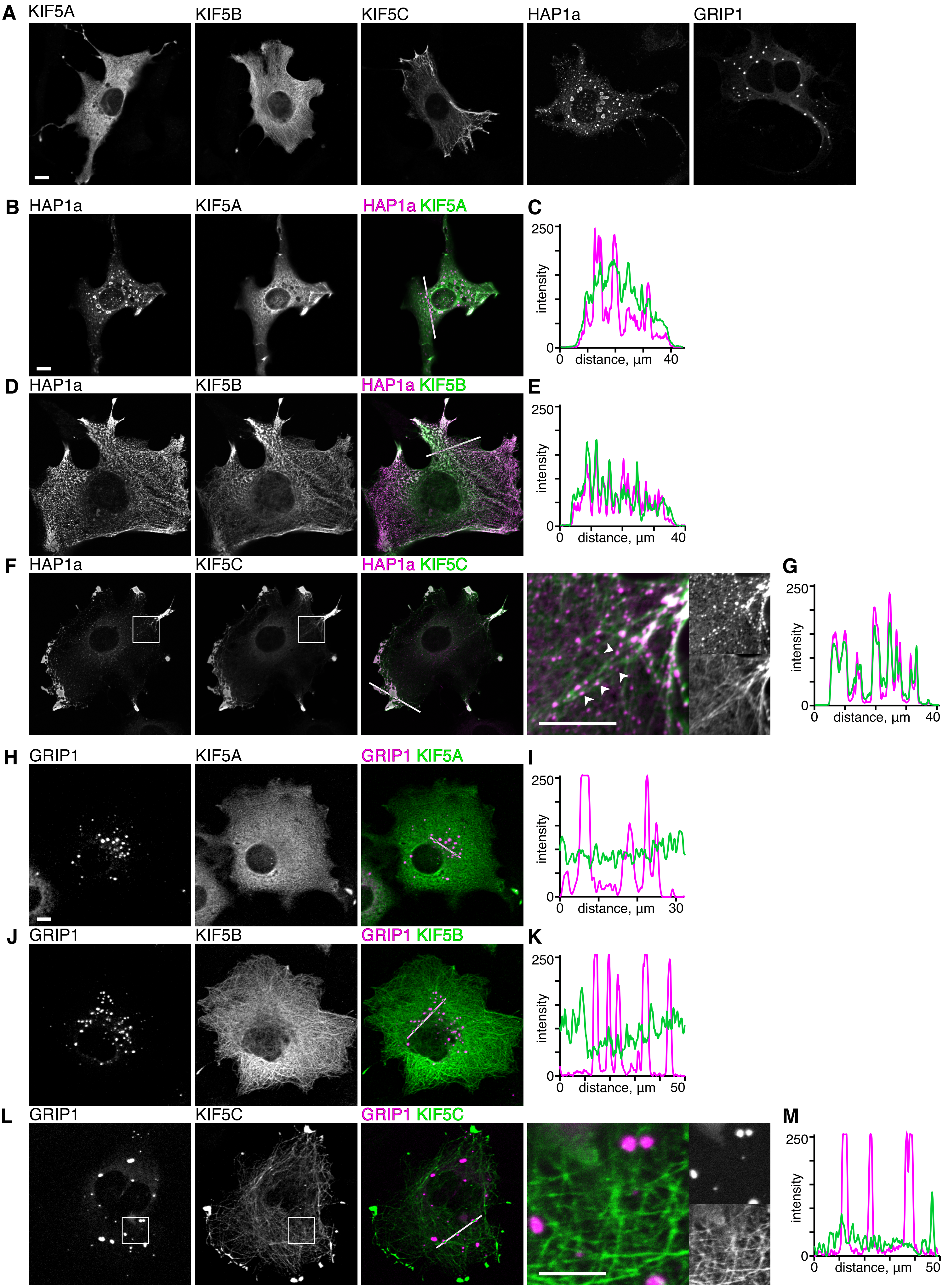

Journal of Cell Science • Supplementary information

Figure S2

HAP1a but not GRIP1 redistributes to the periphery of COS cells with KIF5B and KIF5C, but not KIF5A

A Singly transfected COS cells showing the typical distribution of KIF5A, KIF5B, KIF5C, HAP1a and GRIP1 respectively.

B-G KIF5B and KIF5C, but not KIF5A, recruits HAP1a to the periphery of COS cells. COS cells were transfected with HAP1a-HA and KIF5A-Myc (B) or KIF5B-Myc (D) or KIF5C-Myc (F) and fixed in methanol before staining with rabbit anti-Myc (green) and mouse anti-HA (magenta). Overlap appears white. Scale bars = 10µm. Line scans (C, E, G) through the merged image demonstrates cellular distribution of the proteins with peaks corresponding to punctate structures; KIF5B and KIF5C, but not KIF5A peaks correspond to HAP1a puncta. Small arrowheads in enlarged area of F show HAP1a/KIF5C puncta aligned along microtubules highlighted by surplus KIF5C.

H-M KIF5A, KIF5B and KIF5C are unable to recruit GRIP1 to the periphery of COS cells. COS cells were transfected with GRIP1-GFP (magenta) and KIF5A-Myc (H) or KIF5B-Myc (J) or KIF5C-Myc (L) and fixed in methanol before staining with rabbit anti-Myc (green). Scale bars = 10µm. Line scans (I, K, M) through the merged images demonstrates cellular distribution of the proteins with peaks corresponding to punctate structures; neither KIF5A, B or C overlap with GRIP1 puncta.

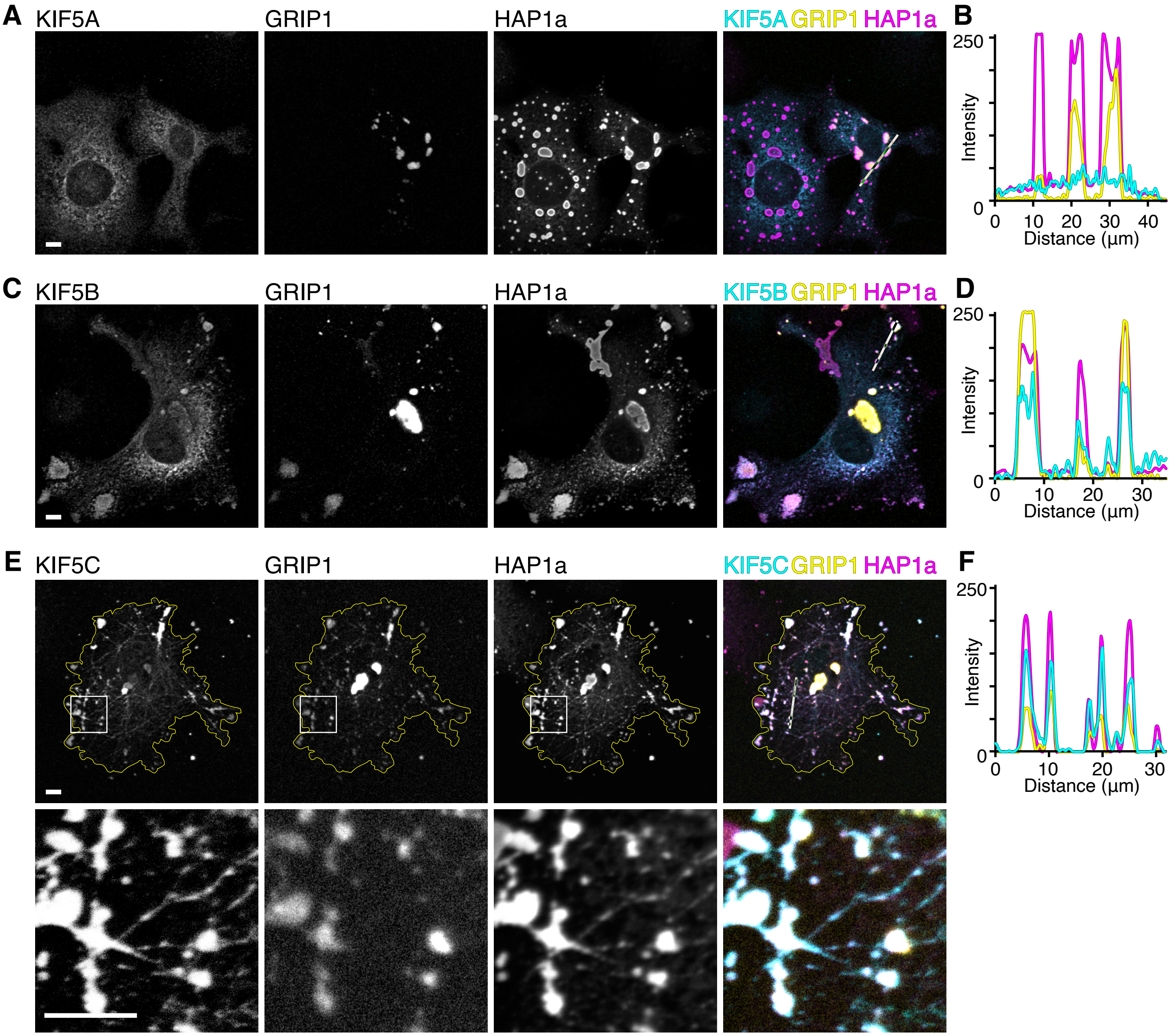

Figure S3

GRIP1 can co-recruit with KIF5B and KIF5C, but not KIF5A, in the presence of HAP1a

A KIF5A is not able to recruit GRIP1 to the periphery of COS cells when HAP1a is also present. COS cells were transfected with HAP1a-HA, GRIP1-GFP (yellow) and KIF5A-Myc and fixed in methanol before staining with rabbit anti-Myc (cyan) and mouse anti-HA (magenta). Scale bar = 10µm.

B Line scan through the merged image demonstrates cellular distribution of the proteins with peaks corresponding to punctate structures. Although GRIP1 and HAP1a localise well with one another, they are not recruited by KIF5A.

C KIF5B is able to recruit GRIP1 to the periphery of COS cells when HAP1a is also present. COS cells were transfected with HAP1a-HA, GRIP1-GFP (yellow) and KIF5B-Myc and fixed in methanol before staining with rabbit anti-Myc (cyan) and mouse anti-HA (magenta). Scale bar = 10µm.

D Line scan through the merged image demonstrates cellular distribution of the proteins with peaks corresponding to punctate structures. All three proteins are localised together in the cell periphery.

E KIF5C is able to recruit GRIP1 to the periphery of COS cells when HAP1a is also present. COS cells were transfected with HAP1a-HA, GRIP1-GFP (yellow) and KIF5C-Myc and fixed in methanol before staining with rabbit anti-Myc (cyan) and mouse anti-HA (magenta). Scale bars = 10µm, cell periphery = yellow line. Boxed area is enlarged region shown on bottom row.

F Line scan through the merged image demonstrates cellular distribution of the proteins with peaks corresponding to punctate structures. All three proteins are localised together in the cell periphery.

Journal of Cell Science • Supplementary information

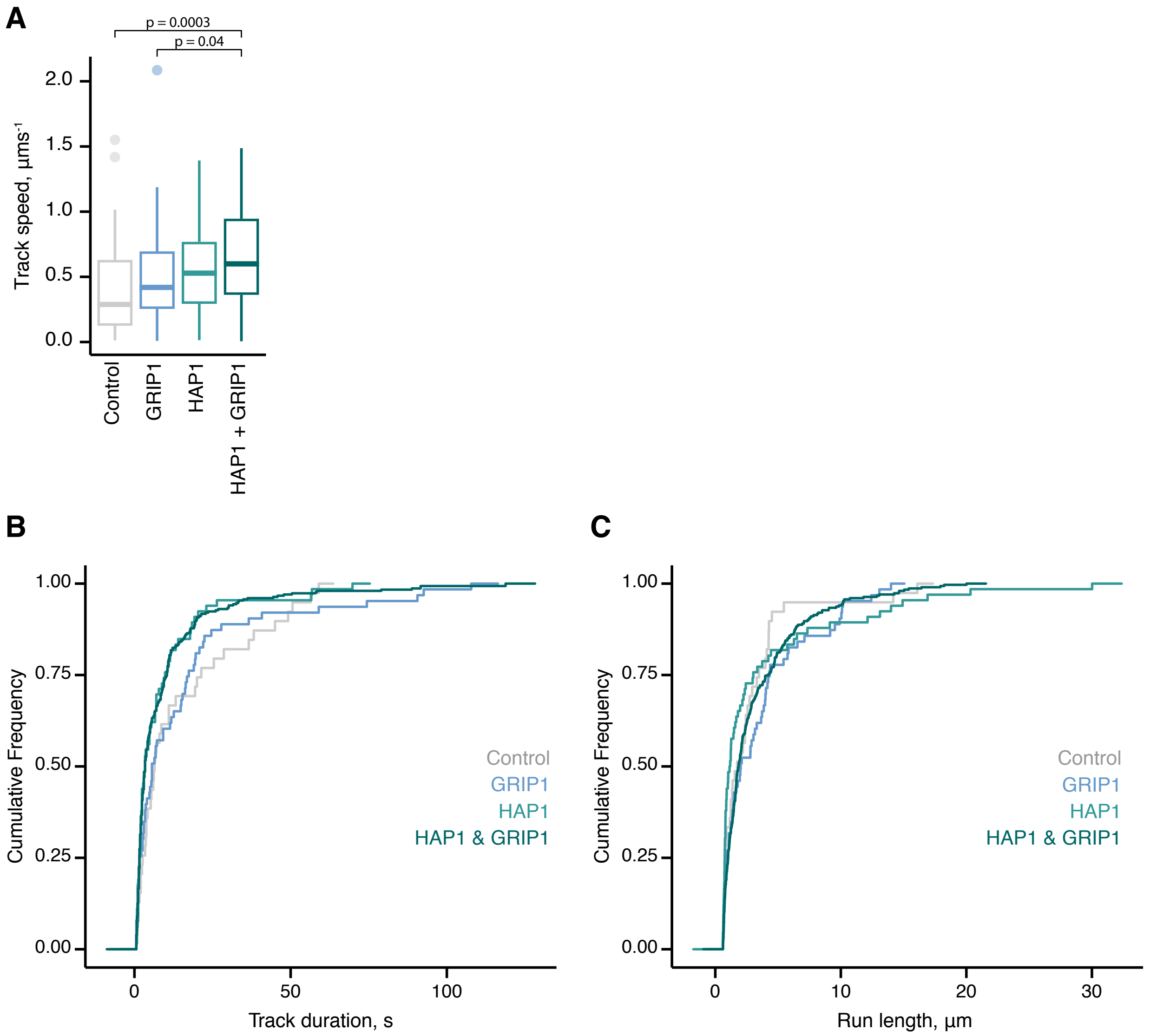

Figure S4

Activation of KIF5 motility in vitro requires the addition of both HAP1a and GRIP1

A Boxplots showing the speed of kinesin tracks in each condition. Median speed: Control, 0.29 µms-1; GRIP1, 0.42 µms-1; HAP1a, 0.53 µms-1; HAP1a & GRIP1, 0.60 µms-1. n = 39, 63, 66 and 302 for Control, GRIP1, HAP1a and HAP1a & GRIP1 respectively from 4 independent experiments.

B Cumulative frequency plot of Track duration (s).

C Cumulative frequency plot of Run length (µm). A comparison between conditions showed no difference between run lengths, likely in part due to a limited number of Control runs skewed by small numbers of endogenous activation events in this system.

Journal of Cell Science • Supplementary information

Supplementary Tables

Table S1

Amplitude (λ) and standard deviation (σ) for three fitted gaussians constrained around the number of bleaching events (μ) corresponding to 2, 4 or 8 GFP molecules.

Bleaching events	GFP-HAP1a Myc-GRIP1a		HA-HAP1a GFP-GRIP1a		GFP-HAP1a GFP-GRIP1a	
**μ**	**λ**	**σ**	**λ**	**σ**	**λ**	**σ**
2	0.69	0.66	1	0.77	0.21	0.63
4	0.31	1.54	0	1.60	0.66	1.13
8	0.00	2.79	0	5.84	0.13	0.98

Journal of Cell Science • Supplementary information
